# Simple defocus laser irradiation to suppress self-absorption in laser-induced breakdown spectroscopy (LIBS)

**DOI:** 10.1016/j.heliyon.2022.e10057

**Published:** 2022-08-01

**Authors:** Alion Mangasi Marpaung, Edward Harefa, Marincan Pardede, Indra Karnadi, Rinda Hedwig, Ivan Tanra, Maria Margaretha Suliyanti, Zener Sukra Lie, Muhandis Shiddiq, Muliadi Ramli, Kurnia Lahna, Eric Jobiliong, Syahrun Nur Abdulmadjid, Nasrullah Idris, Ali Khumaeni, Wahyu Setiabudi, Hery Suyanto, Tjung Jie Lie, Koo Hendrik Kurniawan, Kiichiro Kagawa

**Affiliations:** a Faculty of Mathematics and Natural Sciences, Jakarta State University, Jakarta, 13220, Indonesia; b Key Laboratory of Optical Information Detection and Display Technology of Zhejiang, Zhejiang Normal University, Jinhua 321004, China; c Department of Electrical Engineering, University of Pelita Harapan, Tangerang, 15811, Indonesia; d Department of Electrical Engineering, Krida Wacana Christian University, Jakarta, 11470, Indonesia; e Computer Engineering Department, Faculty of Engineering, Bina Nusantara University, Jakarta, 11480, Indonesia; f Research Center for Physics, Indonesia Institute of Science, Kompleks Puspiptek, Tangerang Selatan 15314, Indonesia; g Automotive & Robotics Program, Computer Engineering Department, Binus ASO School of Engineering, Bina Nusantara University, Jakarta, 11480, Indonesia; h Chemistry Department, Faculty of Mathematics and Natural Sciences, Syiah Kuala University, Darussalam, Banda Aceh, 23111, Indonesia; i Physics Department, Faculty of Mathematics and Natural Sciences, Syiah Kuala University, Darussalam, Banda Aceh, 23111, Indonesia; j Department of Physics, Faculty of Mathematics and Natural Sciences, Diponegoro University, Semarang, 50275, Indonesia; k Department of Physics, Faculty of Mathematics and Natural Sciences, Udayana University, Denpasar, 80361, Indonesia; l Research Center of Maju Makmur Mandiri Foundation, Jakarta, 11630, Indonesia; m Fukui Science Education Academy, Takagi Chuo 2 Chome, Fukui, 910-0804, Japan

**Keywords:** Self-reversal emission lines, Self-absorption, Resonant lines, Defocus laser irradiation, LIBS

## Abstract

This study introduces a novel and simple way to suppress the self-absorption effect in laser-induced breakdown spectroscopy (LIBS) by utilizing a defocusing laser irradiation technique. For this purpose, a Nd:YAG laser with a wavelength of 1,064 nm and repetition rate of 10 Hz with energy in the range of 10 mJ–50 mJ was used. The laser irradiation was focused by using a 150-mm-focal-length plano-convex lens onto the sample surface under defocusing of approximately –6 mm. Potassium chloride (KCl) and sodium chloride (NaCl) pellet samples were used to demonstrate this achievement. When the defocus position is adjusted to –6 mm for KCl and NaCl samples, the self-reversal in the emission lines of K I 766.4 nm, K I 769.9 nm, Na I 588.9 nm, and Na I 589.5 nm vanish. Meanwhile, the FWHM values of K I 766.4 and K I 769.9 nm are 0.29 nm and 0.23 nm, respectively, during –6 mm defocus laser irradiation, as opposed to 1.24 nm and 0.86 nm under tight focus laser irradiation. Additionally, this work demonstrates that, when the laser energy is changed between 10 and 50 mJ, no self-reversal occurs in the emission lines when –6 mm defocus laser irradiation is applied. Finally, a linear calibration curve was generated using KCl at a high concentration ranging between K concentrations from 16.6% to 29%. It should be noted that, even at such high K concentrations, the calibration curve is still linear. This means that self-absorption is almost negligible. This simple change in defocus laser irradiation will undoubtedly contribute to the suppression of the self-absorption phenomenon, which disrupts LIBS analytical results.

## 1 Introduction

Laser-induced breakdown spectroscopy (LIBS) as an analytical technology continues to pique the interest of scientists worldwide. LIBS research has primarily taken two paths since its discovery in the early 1980s. The first concerns the basic aspects of the LIBS plasma, such as plasma dynamics, electron number density, plasma temperature, line broadening and line shift, and plasma modeling [1, 2, 3, 4, 5, 6, 7, 8, 9, 10]. The second is primarily concerned with its numerous applications, which include qualitative and quantitative material analysis [11, 12, 13, 14, 15, 16], biological applications such as clinical specimens and human blood [17, 18], surface analysis including depth profile measurement [19, 20, 21, 22], plant analysis [23, 24, 25], isotopic analysis [26, 27, 28, 29, 30], and many other interesting LIBS applications that are not discussed in detail in this paper.

Despite its popularity as a versatile analytical tool, LIBS still suffers from the self-absorption effect, especially for emission lines originating from direct resonant transitions involving the ground state. The existence of this effect causes the failure to obtain a linear calibration curve, which is needed for reliable quantitative analysis. Many researchers have proposed excellent techniques to overcome or minimize this problem. Hai et al. [31] applied an argon atmosphere and selected the time window for the detection system to minimize self-absorption. Xiong et al. [32] used a fiber laser to suppress the self-absorption in Mg and Ca emission lines. Tang et al. [33, 34] found that a detection window of 0.2–0.4 μs can produce a linear calibration curve for Mn. They also proposed using microwave-assisted excitation in LIBS (MAE-LIBS) to reduce the effect of self-absorption over a wide spectral range of 200–900 nm. Another impressive work, performed by Li et al. [35] employing LIBS assisted by laser-stimulated absorption (LSA-LIBS), can significantly reduce the self-absorption in K, Mn, and Al. Another approach to suppress the effect of self-absorption by using a mathematical model was carried out by Cristoforetti et al. [36], Aragon et al. [37], and Safi et al. [38]. However, their mathematical complexity limited the application. There are many more studies on how to suppress the effect of self-absorption in LIBS that cannot be mentioned individually in this paper, but one can find the references that are mentioned in this paper [39, 40, 41, 42, 43, 44].

In our previous work, to suppress the self-absorption effect in LIBS, we proposed the use of an He metastable excited state to excite the ablated target atoms [45]. The elements K, Na, and Al emit free self-reversal emission lines. For those elements, a linear calibration curve was also obtained. Furthermore, we developed a double pulse orthogonal approach, with which we were able to create emission lines with high concentrations of K, Na, and Al that were practically free of self-reversal and only moderately impacted by the self-absorption phenomenon [46]. Similarly, we recently proposed parallel laser irradiation to minimize the self-absorption phenomenon, and we were able to achieve emission lines of K that were practically free of self-reversal [47].

Although all of the techniques described above produce good results for suppressing self-absorption in LIBS, they are all faced with a significant amount of complexity, including the need to use two lasers, the use of a time-resolved measurement that will reduce the emission intensity to the point where the S/N becomes poor, and the use of a mathematical model to compensate for the self-absorption effect. To address this, we present a novel method for suppressing the self-absorption effect that is both easy and inexpensive, namely, defocusing laser irradiation. Experiments with KCl and NaCl samples revealed free self-reversal emission lines of K I 766.4 nm, K I 769.8 nm, Na I 588.9 nm, and Na I 589.5 nm. Furthermore, even at very high concentrations of KCl, we achieved a linear calibration curve for K, even though we all know that the higher the element concentration is, the greater the self-absorption effect.

## 2 Material and methods

**Figure 1:**
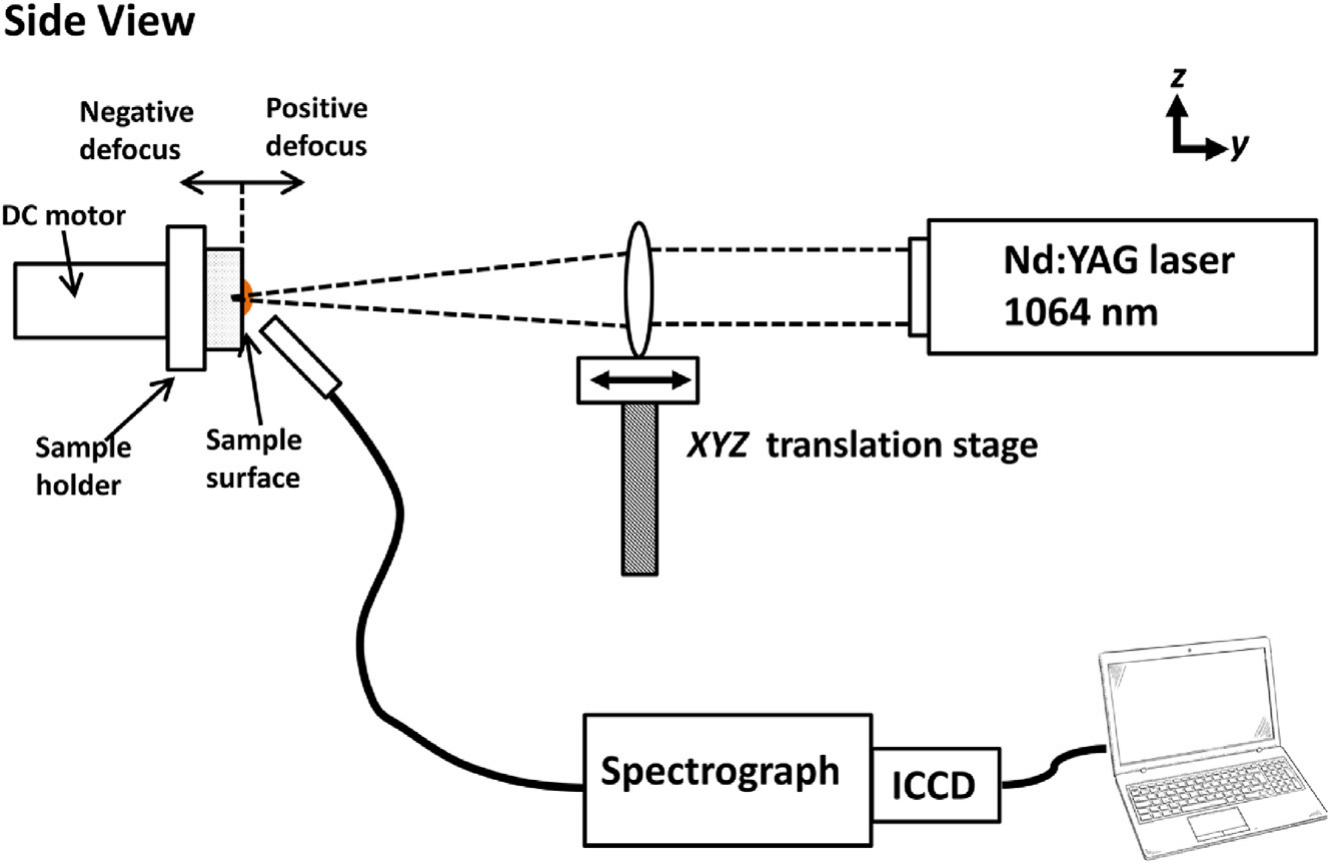
Diagram of the experimental setup.

This experiment used KCl and NaCl powder from Wako Chemical, Japan, with a purity of 4N. Pellets are formed by pressing the powders under 30 MPa pressure for 90 s after they have been ground to an approximately 50 μm grain size. The diameter of the final pellet was 10 mm, while the thickness was 2 mm Figure 1 depicts the experimental setup employed in this study. A fundamental Nd:YAG laser (Quanta Ray model LAB 130–10) is utilized in this experiment, which can operate between 9 to 50 mJ by using a neutral density filter. In all of the trials, the repetition rate of the laser was set to 10 Hz. A plano-convex quartz lens with a 150-mm focal length is utilized to focus the laser irradiation onto the sample surface. The lens is placed above the x-y-z translation stage with micrometer adjustment.

The sample is placed in a sample holder that is attached perpendicular to the servo motor's axis. The servo motor's speed is set to 6 rpm so that each laser irradiation attacks a different location on the sample surface. The entire emission of the created atmospheric plasma is collected using an optical fiber with a core diameter of 50 μm. The fiber is placed approximately 20 mm away from the center of the plasma. The angle between the fiber axis and the laser beam is set to 45°. The opposite end of the optical fiber is attached to the input slit of a spectrograph (McPherson model 2061, focal length 1000 mm, f/8.6, 1,200 g/mm^2^, Czerny Turner configuration), the output slit of which is tied to a gated intensified charge-coupled device (ICCD; Andor iStar intensified CCD, 1,024 × 256 pixels, UK). The synchronized Q-switched laser output is used to trigger the ICCD. The gate delay and gate width of the ICCD are fixed at 1 μs and 30 μs, respectively, making it almost a time-integrated measurement. Under this condition, we can obtain the maximum signal intensity and open the possibility of using the time-integrated CCD system, which is much more compact and cheaper for in situ analysis in the near future. All of the data produced in this work are the average of 20 accumulations of laser irradiation onto the sample surface.

## 3 Results and discussion

Because this study is mainly concerned with the focusing position of the laser irradiation, it is very important to know exactly where the tight focus position is. In this case, we use a pure copper plate (Rare Metallic, 5N, 1 cm in diameter, and a thickness of 0.4 mm) as a sample. We detected the emission spectra of typical Cu emission lines of Cu I 510.5 nm, Cu I 515.3 nm, and Cu I 521.8 nm as the laser energy decreased to as low as 0.5 mJ. By adjusting the y-direction of the x-y-z stage in which the lens is positioned, we determined the highest emission intensities of the above three Cu lines. This position is then marked as a tight focus position (zero position). It is important to note that shifting 0.1 mm away from the sample surface from the tight focus position yielded almost no emission of the above three Cu lines. From this result, we conclude that the precision of the focusing apparatus used in this study is 0.1 mm. We also noted by our naked eyes that the tiny green plasma of Cu disappears when the focusing position is shifted 0.1 mm from its tight focus position. This is mainly due to the very small energy of the laser irradiation used to determine the tight focus position yielding a very high accuracy of the tight focus position.

**Figure 2:**
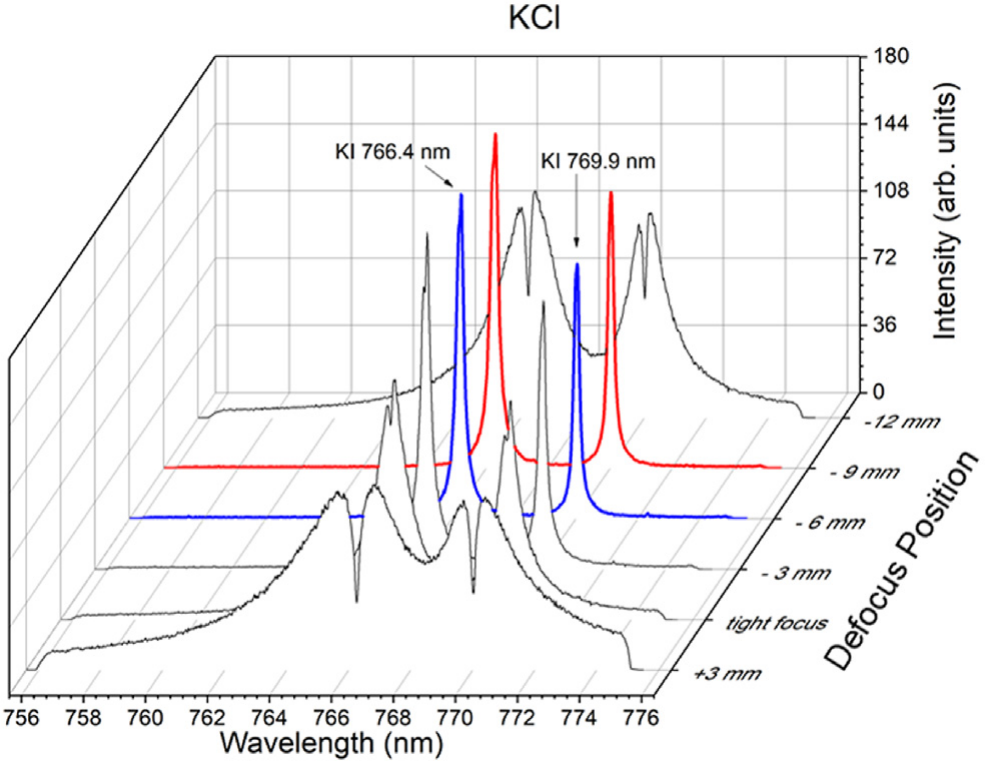
The emission spectra of KCl taken at different focusing positions, tight focus, +3 mm, –3 mm, –6 mm, –9 mm, and –12 mm—denotes a shorter focusing position, and + denotes a longer focusing position. The laser energy is fixed at 21 mJ, and the gate delay and gate width of the ICCD are set to 1 μs and 30 μs, respectively.

**Figure 3:**
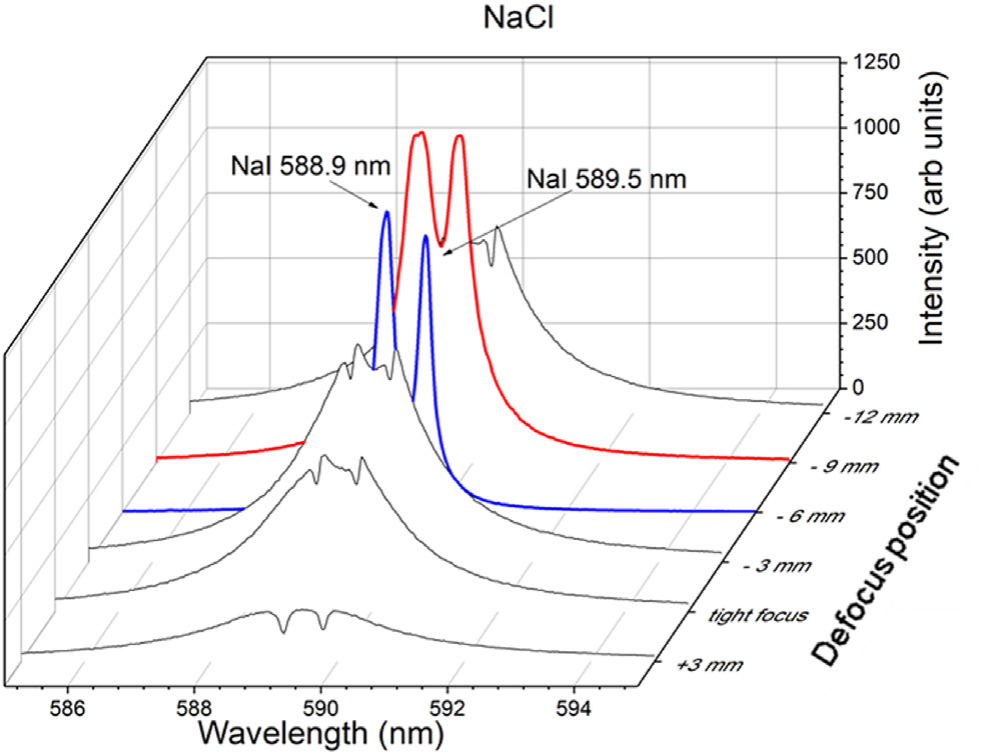
The emission spectra of NaCl taken at different focusing positions, tight focus, +3 mm, –3 mm, –6 mm, –9 mm, and –12 mm—denotes a shorter focusing position, and + denotes a longer focusing position. The laser energy is fixed at 21 mJ, and the gate delay and gate width of the ICCD are set to 1 μs and 30 μs, respectively.

Figure 2 shows the emission spectrum of KCl at various focusing positions, including tight focus, +3 mm, –3 mm, –6 mm, –9 mm, and –12 mm. The negative (–) and positive (+) signs denote a position deviation from the tight focus (zero) position in the y-axis, respectively. The laser energy is fixed at 21 mJ, and the gate delay and width of the ICCD are set to 1 μs and 30 μs, respectively. When laser irradiation is performed at a tight focus, a self-reversing emission spectrum of K I 766.4 nm and K I 769.9 nm is observed, and this effect is exacerbated when the focus position is set to +3 mm. When the focus position is shifted to the negative direction (shorter than the lens's focal length), the self-reversal emission is significantly reduced. At defocus –3 mm, negligible self-reversal of the K I 769.9 nm emission line is observed, but a small self-reversal emission line of K I 766.4 nm is still observed. At defocuses of –6 mm and –9 mm, both K emission lines exhibit a free self-reversal emission line. Further defocus to –12 mm results in significant self-reversal emission of both K emission lines, as well as a significant reduction in emission intensity. We observed that the best emission spectra for K are observed at –6 mm and –9 mm defocus positions. A similar spectrum is obtained for a NaCl sample, as shown in Figure 3. The difference between the NaCl and KCl spectra is minimal, with the best focusing position being only –6 mm. According to the results of Figures 2 and 3, the next experiment will focus exclusively on the –6 mm defocusing position.

The reason why the self-reversal effect is greatly reduced in the negative defocus position could be that, in the defocus position, the density of the plasma is smaller than in the tight focus position. However, when there is too much negative defocus, such as –12 mm, the fluence of the laser becomes too small, resulting in a serious self-reversal process. This explanation also applies to +3 mm defocus because most of the laser energy is used to create air breakdown in front of the sample surface and only a small fraction of the laser energy is used to ablate the sample. We also observe that the beam waist diameter is 25.4 μm for tight focus and 141 μm for defocus at—6 mm. Meanwhile, the fluence (intensity) is 0.41 × 10^4^ J/cm^2^ (0.52 × 10^12^ W/cm^2^) at tight focus and 0.13 × 10^3^ J/cm^2^ (0.17 × 10^11^ W/cm^2^) for defocus at –6 mm.

To strengthen our assumption that a lower plasma density can suppress the self-absorption effect, an additional experiment was carried out in the same way as in Figures 2 and 3. However, this time, the H_α_ line (H I 656.2 nm) spectra are taken, and the full width at half maximum (FWHM) measurements are found to be 1.67 nm for the tight focus position and 1.25 nm for the defocus –6 mm position. This means that, at the defocus position, the density of the plasma is lower than at the tight focus position.

**Figure 4:**
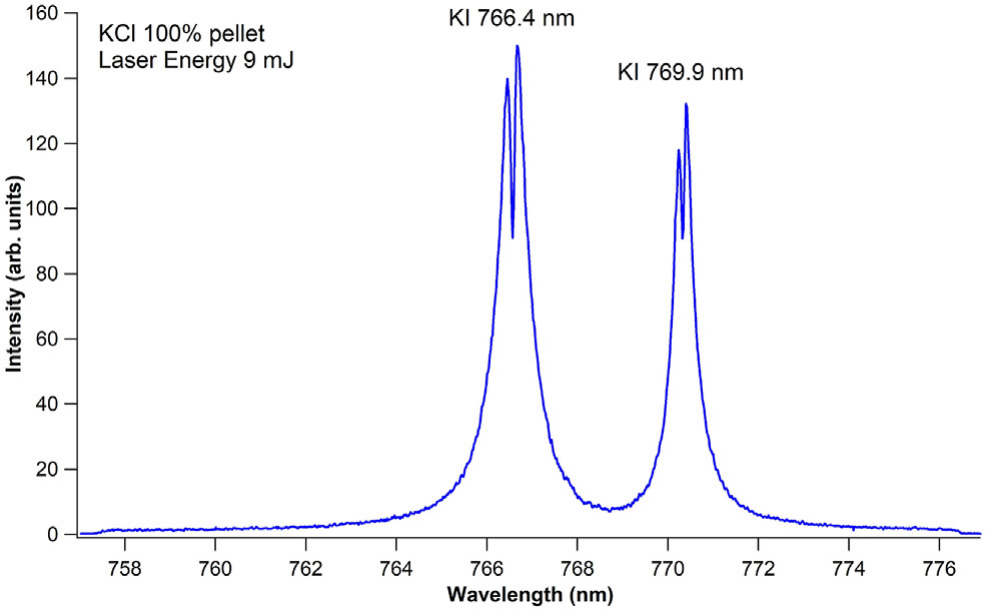
Emission spectra of the KCl pellet under 9 mJ laser energy. The focusing position is set at tight focus. The gate delay and gate width of the ICCD are set to 1 μs and 30 μs, respectively.

One might also assume that the suppression of the self-absorption effect observed in the defocus –6 mm condition is due to the low laser fluence, which may also be achieved in the tight focus condition by lowering the laser energy. However, when the laser energy in the tight-focus position is reduced to 9 mJ, the self-reversal is still clearly visible, as shown in Figure 4. This suggests that suppressing the self-absorption effect with a tight focus is difficult.

**Figure 5:**
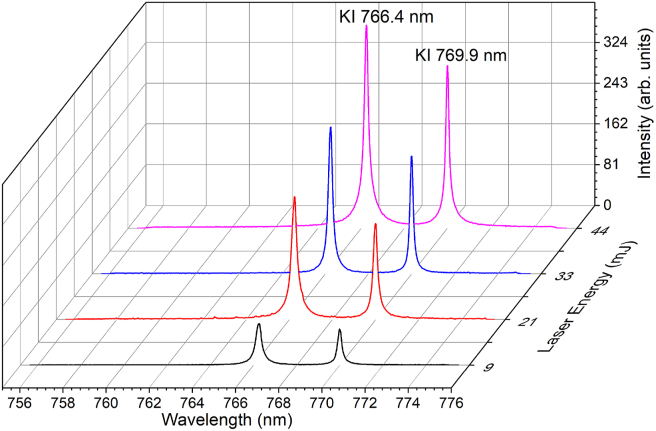
Emission spectra of the KCl pellet under different laser energies. The focusing position is set at a defocus of –6 mm. The gate delay and gate width of the ICCD are set to 1 μs and 30 μs, respectively.

To better understand the dependence of the laser energy at the defocusing point of –6 mm, the KCl spectra were collected again, and the results are given in Figure 5. Once again, the –6 mm defocus position provides the best spectral results without requiring a self-reversal process for the various energy lasers used. One might wonder why we only use laser energies ranging from 10 to 50 mJ. This is because we hope to use this technique for in situ analysis soon, and in that case, a small portable Nd:YAG laser with a maximum energy of 50 mJ is easily available on the market.

**Figure 6:**
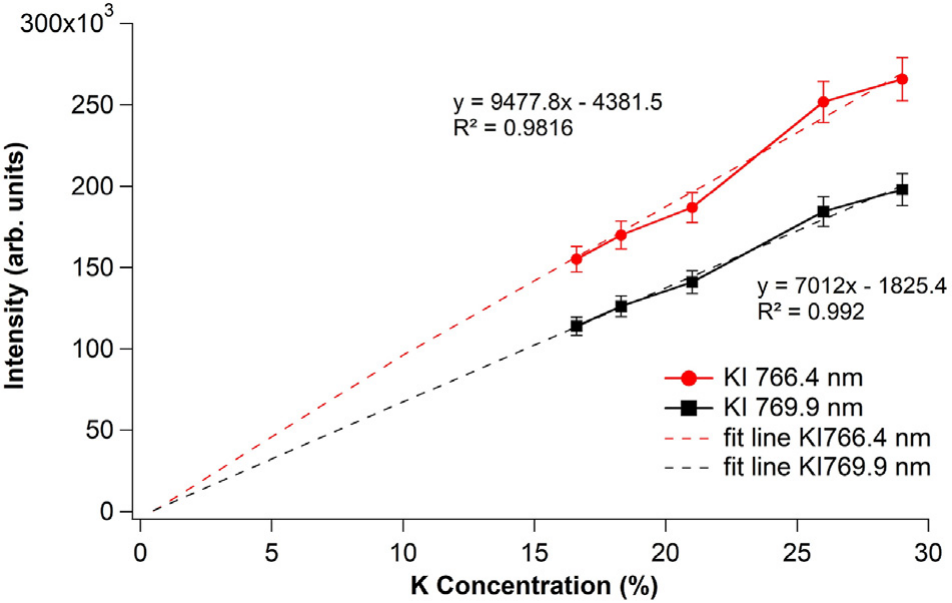
Plot between the emission intensity of K I 766.4 nm and K I 769.9 nm as a function of K concentration. Each data point in this figure is obtained from the average of 5 data points produced by 20 successive laser irradiations each.

Finally, various pellet samples with different concentrations of KCl were prepared to determine the linearity of the calibration curve. NaCl is used as a KCl companion. Figure 6 shows the KCl calibration curves at 30%, 35%, 40%, 50%, and 55% concentrations, which corresponds to K concentrations of 16.6%, 18.3%, 21%, 26% and 29%, respectively. In Figure 6, the measured K I 766.4 nm and K I 769.9 nm emission intensities are plotted against the associated K concentrations. Each data point in this graph is the average of five data points obtained from 20 successive laser irradiations. Over the dynamic range used in this study, the K concentration and its associated emission intensity show a clear linear relationship with a very high determination coefficient R^2^ of 0.99 for K I 769.9 nm and 0.98 for K I 766.4 nm. This result demonstrates that our novel defocus laser irradiation technique is effective in suppressing self-absorption in LIBS.

## 4 Conclusion

In conclusion, the laser irradiation defocus method developed in this study can suppress the effect of self-absorption on LIBS. The linear calibration curve of KCl at high concentrations demonstrates this. It should also be noted that the spectrum we show is a time-integrated spectrum, which means that a quantitative analysis of the self-absorption-free LIBS method can be performed in field studies by combining a portable laser and a time-integrated CCD, both of which are widely available on the market.

## Declarations

### Author contribution statement

Alion Mangasi Marpaung; Kiichiro Kagawa; Indra Karnadi: Conceived and designed the experiments.

Koo Hendrik Kurniawan: Conceived and designed the experiments; Wrote the paper.

Edward Harefa; Marincan Pardede; Ivan Tanra; Maria Margaretha Suliyanti: Performed the experiments.

Zener Sukra Lie; Muhandis Shiddiq; Muliadi Ramli; Kurnia Lahna; Eric Jobiliong; Syahrun Nur Abdulmadjid: Analyzed and interpreted the data.

Nasrullah Idris; Ali Khumaeni; Wahyu Setiabudi; Hery Suyanto; Tjung Jie Lie: Contributed materials and analysis tools.

### Funding statement

Dr. Koo Hendrik KURNIAWAN was supported by Third World Academy of Sciences [060150 RG/PHYS/AS/UNESCO FR:3240144882].

Alion Mangasi Marpaung was supported by Kementerian Riset, Teknologi dan Pendidikan Tinggi [NO:7/E/KPT/2019 and NO:22/SP2H/DRPM/LPPM-UNJ/III/2019].

### Data availability statement

Data will be made available on request.

### Declaration of interest's statement

The authors declare no conflict of interest.

### Additional information

No additional information is available for this paper.
